# Removal of Nutrients From Anaerobically Digested Swine Wastewater Using an Intermittent Cycle Extended Aeration System

**DOI:** 10.3389/fmicb.2020.576438

**Published:** 2020-10-16

**Authors:** Nguyen Hong Dan, Eldon R. Rene, Tran Le Luu

**Affiliations:** ^1^Department of Chemical Engineering, Nong Lam University, Ho Chi Minh City, Vietnam; ^2^Department of Environmental Engineering and Water Technology, IHE Delft Institute for Water Education, Delft, Netherlands; ^3^Master Program in Water Program Technology, Reuse and Management, Vietnamese German University, Thu Dau Mot, Vietnam

**Keywords:** swine wastewater, intermittent cycle extended aeration system, nutrient, biological, anaerobic

## Abstract

Swine wastewater contains high concentrations of organic compounds, nutrients (nitrogen and phosphorus), heavy metals, and residual antibiotics, amongst others, that have negative impacts on the water environment. The main aim of this work was to remove nutrients from anaerobically digested swine wastewater using an intermittent cycle extended aeration system (ICEAS). The effects of operational parameters such as cycle time, organic loading rate, C/N ratio, and aeration/mixing ratio on the pollutant removal efficiencies of ICEAS were studied and compared with the performance of a conventional sequencing batch reactor (SBR). The following optimal conditions were obtained: cycle time, 6 h; organic loading rate, 0.86 kg COD m^−3^ day^−1^; C/N ratio, 2.49–2.82; and aeration/mixing ratio, 1.57. The pH was maintained in the range of 6.0–8.0. The total organic carbon (TOC), total nitrogen (TN), ammonium (NH_4_^+^), total phosphorus (TP), and color removal efficiencies of ICEAS were higher than those of the conventional SBR, with removal efficiencies of 95.22, 88.29, 97.69, 85.81, and 97.84%, respectively, compared to 94.34, 81.16, 94.15, 77.94, and 96.95%, respectively, observed in the SBR. TOC, TN, NH_4_^+^, TP, and the color removal efficiencies of ICEAS were higher by 0.88, 7.13, 3.54, 7.87, and 0.95%, respectively, than the conventional SBR. The good results from this study show that ICEAS is a promising technology for the removal of organic contaminants and nutrients from anaerobically digested swine wastewater and that the effluent water quality meets the Vietnamese discharge standard (QCVN 62-MT:2016/BTNMT) for swine wastewater effluents.

## Introduction

The livestock sector, especially the swine industry, plays an important role in promoting agricultural development and the economy of a country (Shin et al., 2005). The swine wastewater generated from pig farms can cause a negative effect on the water environment when discharged without adequate treatment. Swine wastewater is a mixture of pig urine, floor washing water, sediment, and fecal matter (Sakar et al., 2009). It contains high concentrations of many toxic compounds such as organic substances, nitrogen, phosphorous, and residual antibiotics (Zhou et al., 2007; Rajagopal et al., 2011; Richardson and Ternes, 2011; Li et al., 2012; Wegst-Uhrich et al., 2014; Bailey et al., 2016). Anaerobic digestion (AD) of swine wastewater can convert organic compounds to biogas and renewable energy (Sui et al., 2014). AD offers the following advantages: (i) treatment of high-strength wastewater, (ii) high conversion of chemical oxygen demand (COD) to biogas, (iii) ease of maintenance, (iv) good process control, (v) ability to tolerate fluctuating COD loads, i.e., feast and famine conditions, and (vi) good stability of the anaerobic biomass. Nevertheless, this technique produces large amounts of anaerobically digested effluent that contains nutrients, inorganic salts, organic compounds (amino acid and B vitamins), and trace/heavy metals (Fe, Cu, and Zn). This effluent, when left untreated, affects the water environment and causes problems such as the eutrophication of rivers and lakes and odor problems (Wen et al., 2016). Besides that, the emission of unpleasant smell during the anaerobic process of swine wastewater may induce air pollution that brings about adverse impacts on human health. Therefore, the development of a new technology to treat anaerobically digested swine wastewater is urgently required to satisfy the environmental discharge regulations (An et al., 2007; Daumer et al., 2007; Dosta et al., 2008).

Several physicochemical and biological technologies have been used to treat swine wastewater, namely, electrocoagulation (Mores et al., 2016), electrochemical (Huang et al., 2016, 2018) and biological methods such as anaerobic-aerobic (Aziza et al., 2019; Shoukat et al., 2019), moving bed biofilm reactor (MBBR; Yang et al., 2015), and membrane bioreactor (MBR; Guglielmi and Andreottola, 2011; Guadie et al., 2014). However, these technologies have limitations such as high initial investment cost, consumption of large quantities of chemicals, requiring large footprint, complex operation and control of parameters, and low nitrogen and phosphorous removal efficiencies. Typically, most of the large- (>1,000 pigs) and small-scale (<100 pigs) pig farms in Vietnam are using the AD process for swine wastewater treatment (Sakar et al., 2009). However, the anaerobically digested swine wastewater does not meet the Vietnamese discharge standard for swine wastewater effluent QCVN 62-MT:2016/BTNMT, especially for COD, biochemical oxygen demand (BOD_5_), and NH_4_^+^. The treated water cannot be reused, causing waste of water and environmental pollution. Therefore, it is necessary to adopt a suitable treatment technology to treat this wastewater, minimize the environmental pollution, and build a water reuse cycle in pig farms. This is one of the main driving forces behind this practically relevant case study.

Recently, the intermittent cycle extended aeration system (ICEAS) technology has been widely applied for the treatment of many types of wastewaters because it offers the following advantages: (i) small footprint, (ii) low energy consumption, (iii) good process control and sequenced operational cycles, and (iv) high removal of nitrogen and organic pollutants (Li et al., 2008; Zhang et al., 2012). The ICEAS technology is an improved version of the sequencing batch reactor (SBR) technology, with continuously flowing influent, and it is fully automatic (Coelho et al., 2000; Fikar et al., 2005; Spagni and Marsili-Libelli, 2009). Due to its mode of operation and design, its advantages include: (i) reduced consumption of dissolved oxygen (DO), (ii) less energy requirement, i.e., 10–15% less energy for aeration compared to aeration-based biological processes (Liu and Wang, 2017; Sanchez et al., 2018), (iii) ability to tolerate shock loads of organics, (iv) reduced emission of greenhouse gas (~36%), and (v) high total nitrogen (TN) removal efficiency compared to the SBR technology (Spagni and Marsili-Libelli, 2009). Several previous research works have also reported on the removal of nutrients (nitrogen and phosphorus) from different wastewater sources using ICEAS. For example, Qiu et al. (2019) achieved TN removal of 81.5% during landfill leachate treatment, Zhang et al. (2011) reported TN removal of 76.5% for anaerobically digested swine wastewater, and Al-Rekabi et al. (2017) treated municipal wastewater and reported a total phosphorus (TP) removal efficiency >72%. Moreover, the biodegradability of nitrogen-rich wastewater is high in an ICEAS compared to other traditional technologies, especially for swine wastewater. However, for successful long-term operation, the optimization of the aeration/stirring ratio will reduce the operation time and increase the nitrogen removal efficiency (Mosquera-Corral et al., 2005; Gabarro et al., 2013; Pan et al., 2014a; Zheng et al., 2017). In an ICEAS, all the unit operations and biological reactions occur in the same tank, and the tank is controlled automatically with the help of sensors and actuators, which is suitable for installation and operation in small pig farms.

Besides, ICEAS can be used to treat many different types of wastewaters such as industrial, municipal, and tannery wastewater (Yoong et al., 2000; Maranón et al., 2008; Elmolla and Chaudhuri, 2011; Mojiri et al., 2014; Wang et al., 2015). Bao-Cang et al. (2018) used synthetic wastewater and showed total organic carbon (TOC) and ammonium (NH_4_^+^) removal efficiencies of 81.6 and 92.1%, respectively. The TN and TP removal efficiency of municipal wastewater using ICEAS was ~90% (Liu and Wang, 2017). Goncalves et al. (2005) used wool dyeing wastewater and indicated that the COD and BOD_5_ removal efficiencies were >80%. Li et al. (2008) treated slaughterhouse wastewater using ICEAS and showed that the COD, TN, and TP removal efficiencies were 96 and 99%, respectively. ICEAS has also been applied to treat other specific sources of wastewaters, e.g., landfill leachate treatment (Qiu et al., 2019), municipal wastewater (Xu et al., 2020), and domestic wastewater (Khondabi et al., 2019). The main microbial communities of ICEAS include the following: ammonium-oxidizing bacteria (AOB), e.g., *Nitrosomonas*, *Nitrosococcus*, *Nitrobacter*, and *Nitrococcus* (Koops and Pommerening-Roser, 2001), which play important roles in ammonium oxidation, and nitrogen-oxidizing bacteria (NOB), e.g., *Nitrobacter*, *Nitrococcus*, *Nitrospina*, and *Nitrospira* (Ge et al., 2015), which play important roles in denitrification. It is noteworthy to mention that nitrogen removal in a ICEAS occurs according to the following main mechanisms involving nitrification/denitrification by AOB and NOB (Eq. 1).

(1) $${\mathrm{NH}}_{4}{}^{+}\to {\mathrm{NO}}_{2}{}^{-}\to {\mathrm{NO}}_{3}{}^{-}\to N_{2}$$

Besides, ammonium is converted into N_2_ gas *via* the partial nitrogen process, according to Eq. 2 (Yamamoto et al., 2008; Bournazou et al., 2013; Anjali and Sabumon, 2017; Zheng et al., 2017).

(2) $$\begin{array}{l} {\mathrm{NH}}_{4}{}^{+}+1.31{\mathrm{NO}}_{2}{}^{-}+\\ 0.066{\mathrm{HCO}}_{3}{}^{-}+0.13H^{+}\to 1.02N_{2}+026{\mathrm{NO}}_{3}{}^{-}\\ \;\;\;\;\;\;\;\;\;\;\;\;\;+0.066{\mathrm{CH}}_{2}O_{0.5}N_{0.5}+2.03H_{2}O \end{array}$$

Phosphorus removal is an integral part of a wastewater treatment plant, and in biological treatment systems, phosphorus is normally treated by absorption into cell biomass by polyphosphate-accumulating organisms (PAOs). In a previous study, the phosphorus removal efficiency of ICEAS was reported to be >90%, which is higher than that of the conventional activated sludge technology (~10–20% P removal; Dutta and Sudipta Sarkar, 2015). Nitrogen removal from nitrite occurs under oxygen-limiting conditions (DO ≤ 0.5 mg/L; Guisasola et al., 2005); therefore, the DO concentration in the bioreactor has to be adjusted by optimizing the aeration/mixing rates. This unit operation requires prior operational knowledge of the reactor’s operational modes and good process control instrumentation to control the different parameters of the reactor. Anew, other factors such as the carbon source, reaction time, and pollution load also have adverse impacts on the efficiency of this process. Hence, the main aim of this research was to study the effects of operating conditions such as cycle time, organic loading rate, C/N ratio, and aeration/mixing ratio on the removal of nutrients and organics present in anaerobically digested swine wastewater using the ICEAS technology.

## Materials and Methods

### Swine Wastewater

Swine wastewater was collected at the swine farm of Ms. Nguyen Thi Tin, located in Village 1, Tan Dinh Commune, Ben Cat District, Binh Duong Province, Vietnam “11-0452551; 106-6447753.” The farm has a capacity of 100 swines. Swine wastewater was collected from the anaerobic tank of the existing anaerobic wastewater treatment plant. The swine wastewater before and after anaerobic treatment still contains pollutants that exceed the Vietnamese allowable discharge standard for swine wastewater effluent, i.e., according to the rule QCVN62-MT:2016/BTNMT, as shown in Table 1. The influent swine wastewater was subjected to primary treatment where sand particles, residual solids, and large garbage were removed and the primary treated water was settled for 1 h. The seed activated sludge, i.e., the inoculum, was collected in an anaerobic SBR (ASBR) tank of the domestic wastewater treatment plant of Thu Dau Mot City, Binh Duong, Vietnam. The inoculum was acclimated using swine wastewater for a period of 2 weeks. The sludge volume index (SVI) was kept constant at 3,000 ml/g, and the BOD_5_/COD ratio of the influent swine wastewater was 0.5.

**Table 1:** Properties of raw swine wastewater after anaerobic treatment and after the intermittent cycle extended aeration system (ICEAS).

No.	Parameter	Unit	Raw swine wastewater	After anaerobic treatment	After ICEAS treatment	Vietnamese discharge standard QCVN62-MT:2016/BTNMT (column B)
1	pH	–	6.9	8.4	8.8	5.5–9.0
2	Color	Pt‒Co	4,576.52	4,104.21	88.33	–
3	COD	mg/L	3,459.43	2,267.62	157.78	300
4	BOD_5_	mg/L	2,100.34	1,133.23	13.89	100
5	TN	mg/L	975.45	862.92	96.67	150
6	NH_4_^+^	mg/L	623.86	476.35	10.94	–
7	Nitrate	mg/L	377.43	462.67	50.98	–
9	TOC	mg/L	389.34	341.18	16.30	–
10	TP	mg/L	482.62	415.34	52.46	–

COD, chemical oxygen demand; BOD_5_, biochemical oxygen demand; TN, total nitrogen; TOC, total organic carbon; TP, total phosphorus.

### Experimental System

**Figure 1:**
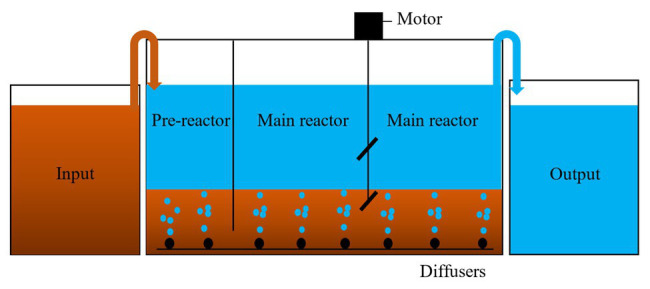
Schematic of the ICEAS system.

A lab-scale ICEAS system was constructed using plastic acrylic material, with dimensions of 500 mm × 200 mm × 450 mm (L × W × H) and with a total working volume of 40 L, as shown in Figure 1. The ICEAS consists of two reaction zones (i.e., the pre-reaction zone and the main-reaction zone) which are connected to each other with the help of a 20-mm bottom space. The pre-reaction zone has dimensions of 100 mm × 200 mm × 450 mm (L × W × H), with a working volume of 8 L, while the main reaction zone has dimensions of 400 mm × 200 mm × 450 mm (L × W × H), with a working volume of 32 L. The pre-reaction zone was stabilized with a continuous flow of the influent in order to limit organic shock load to the microorganisms present in the main reaction zone. The oxygen was supplied using air blower systems and air pumps. The DO concentration was maintained at 2.5 mg/L during the operation of the ICEAS. Activated sludge was stirred using a 380-mm paddled motor. The input swine wastewater was filled into the pre-reaction zone (continuous inflow) by a peristaltic pump and then the wastewater flowed into the main reaction zone *via* the bottom space. Herein, the intermittent aeration process occurred and, finally, the treated water was decanted into the output tank using a pump. The cycle includes four phases of fill, reaction phase (aeration/mixing period), settling phase, and decanting phase. First, an aeration time of 45 min and a mixing time of 15 min, and then the cycle is repeated two times. After the completion of the reaction period, the aeration and mixing process was stopped and settling was done for 60 min. After that, the treated water was decanted for 30 min. The effects of cycle time, organic loading rate, and the aeration/mixing time on the nutrient and organic removal efficiencies were evaluated. The cycle times were varied from 4.5 to 8 h. The organic loading rate was increased from 0.34 to 2.58 kg COD m^−3^ day^−1^, while the aeration/mixing times were varied from 75 to 195 min. Then, the pollutant removal efficiencies of ICEAS were compared with those of the conventional SBR process.

### Wastewater Analysis

All the parameters monitored in this study were determined according to the protocols described in the Standard Methods for the Examination of Water and Wastewater (APHA et al., 2012). Wastewater was withdrawn and collected for the analysis of different pollutants’ concentrations at regular time intervals. The experiments were replicated three times and the average values are shown in this study (Tien and Luu, 2020). The pH, color, and conductivity were measured using a Metrohm 900 multimeter (Switzerland). COD was measured using a Lovibond RD125 Thermoreactor (England), which uses the closed reflux titrimetric method for analysis. The TOC and TN contents were measured using a TOC Shimadzu 00936 (Japan). BOD_5_ was measured using the Winkler method in a strong base environment at 20°C for 5 days using a BOD-System (Lovibond, Germany). The NO_3_^−^, NH_4_^+^, and TP concentrations were determined using ion chromatography [Metrohm IC 883, Switzerland; limit of detection (LOD) ≤ 0.05 mg/L]. The pollutant removal efficiencies were calculated based on the difference between the input and output wastewater concentrations, according to Eq. 3.

(3) $$\mathrm{Removal efficiency}=\frac{\mathrm{ONt}-\mathrm{ONs}}{\mathrm{ONt}}\times 100\;$$

where ONt and ONs (in milligrams per liter) are the concentrations of pollutants in swine wastewater before and after treatment, respectively.

## Results and Discussion

### Effect of Cycle Time

**Figure 2:**
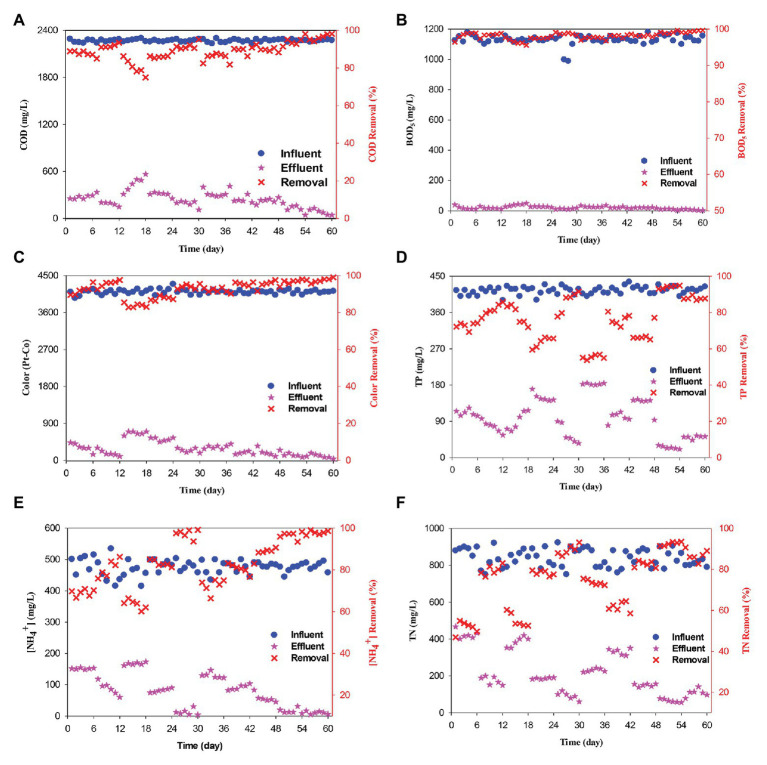
Swine wastewater treatment using the ICEAS at different cycle times, organic loading rates, and aeration/mixing rates. **(A)** COD, **(B)** BOD_5_, **(C)** color, **(D)** TP, **(E)** ammonium (NH_4_^+^‒N), and **(F)** TN removal efficiencies.

The swine wastewater treatment using the ICEAS at different cycle times is shown in Figure 2. The cycle time was changed by changing the mixing time for the 4.5-h cycle (45 min mixing), 6-h cycle (135 min mixing), and 8-h cycle (255 min mixing). The experiment to determine the optimal cycle time was conducted during the first 18 days, wherein the organic loading rate was maintained at 1.71 kg COD m^−3^ day^−1^ in order to support adequate microbial growth without causing substrate-induced inhibition to the microorganisms. Previous reports have also used similar ranges of organic loading rates, e.g., Liang et al. (2019) used a range of 1.8–2.5 kg COD m^−3^ day^−1^. The results show that, at a cycle time of 4.5 h (experiment was conducted from day 1 to 6), the TN, TP, and NH_4_^+^‒N removal efficiencies were not high and were only 51.67, 72.46, and 69.12%, respectively. The next experiment was performed in order to increase the mixing time to 135 min (6-h cycle time). The TN, TP, and ammonium removal efficiencies increased to 79.57, 81.44, and 80.75%, respectively. This can be explained by the fact that increasing the mixing time will increase the denitrification by the partial nitrogen process, leading to an increase in the TN and NH_4_^+^‒N removal efficiencies.

**Figure 3:**
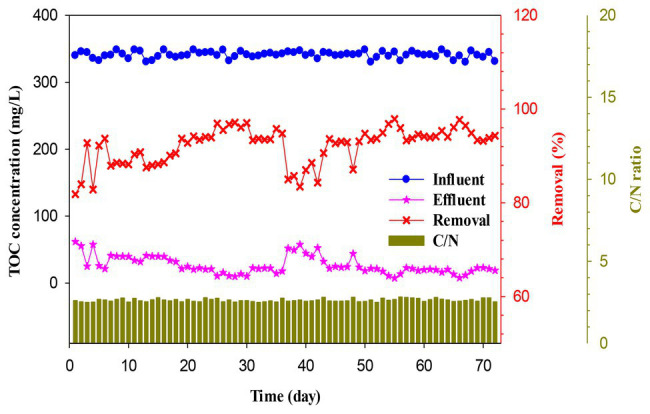
Effect of the C/N ratio on the TOC removal efficiency of the ICEAS.

However, in this study, it was not possible to conclude that the 6-h cycle is optimal for high performance of the reactor. Therefore, the mixing time was increased to 255 min (8-h cycle), between days 13 and 18. The nitrogen and phosphorus removal efficiencies after a cycle time of 8 h significantly decreased compared to those with a cycle time of 6 h. The nitrogen and phosphorus removals mainly occurred in the non-aerated phase (Gao et al., 2013) by the conversion of nitrate (NO_3_^−^) into N_2_ gas and phosphate (PO_4_^3−^) consumption by phosphorus-accumulating bacteria (i.e., for every 1 mg/L of phosphorus consumed, approximately 7.5–10.7 mg/L of COD; Song et al., 2017). Thus, the nitrogen and phosphorus removal efficiencies increased when increasing the non-aerated phase time (mixing time; Akin and Ugurlu, 2004; Melidis, 2014). However, when the mixing time was too long (i.e., the hydraulic retention time increases), the degradation of organic compounds was efficient, although nutrient depletion caused a reduction in the efficiency of microorganisms. A similar observation was previously reported by Melidis (2014) during landfill leachate treatment. The nitrogen and phosphorus removal efficiencies also decreased when the mixing time was 2 h. The TOC and BOD_5_ removal efficiencies in all the three cycles were over 90%, which proves the ability of ICEAS to treat organic compounds. The TN and TP removal efficiencies in all the three cycles were low due to the effect of stirring time. Therefore, it can be concluded that the most optimal cycle time is 6 h. In a previous work, used an 8-h cycle time for treating slaughterhouse wastewater and obtained COD, TN, and TP removal efficiencies >90%. Pan et al. (2014a) showed that treating slaughterhouse wastewater at a cycle time of 12 h in an ICEAS tank reached TN removal efficiency of 42.8%. Kayranli and Ugurlu (2011) indicated that the cycle time of 6 h was best for municipal wastewater treatment. Sheng et al. (2017) treated swine wastewater using ICEAS at a cycle time of 8 h and reported TN and NH_4_^+^ removal efficiencies of 79 and 89%, respectively. Zhang et al. (2011) performed swine wastewater treatment using ICEAS and reported TN removal efficiency of 97% at a cycle time of 8 h. In another recent study, Xu et al. (2020) reported a nitrogen removal efficiency of 89% when operating the ICEAS with a cycle time of 7 h. Furthermore, the authors also indicated that, when the cycle time was increased, an increased nitrate accumulation was noticed in the reactor. Khondabi et al. (2019) applied ICEAS for domestic wastewater treatment, with a cycle time of 8 h, and reported COD and BOD_5_ removals of 93 and 95%, respectively.

### Effect of Organic Loading Rates

**Figure 4:**
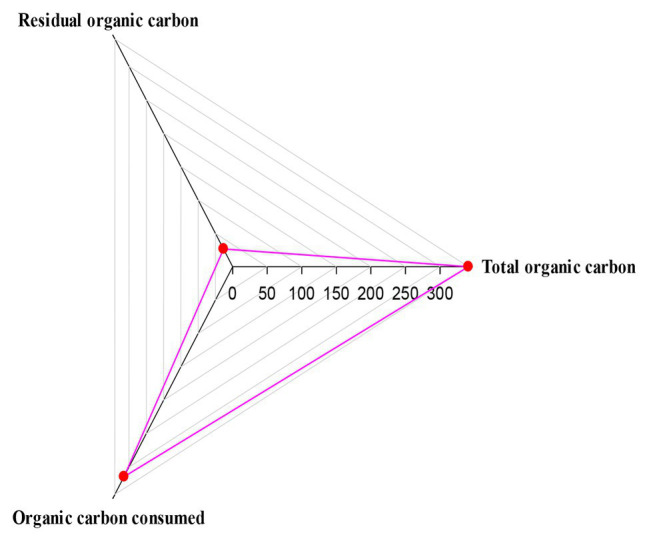
Consumption of organic carbon in the ICEAS reactor.

The effect of organic loading rate was ascertained by performing experiments at different organic loading rates, i.e., 0.34, 0.86, and 2.58 kg COD m^−3^ day^−1^. The organic loading rate was reduced from 1.71 to 0.86 kg COD m^−3^ day^−1^ between days 25 and 30. At an organic loading rate of 0.86 kg COD m^−3^ day^−1^, the COD, BOD_5_, TOC, and color removal efficiencies were 86.37, 97.60, 93.68, and 87.63%, respectively. At this organic loading rate, the TN, TP, and ammonium removal efficiencies were respectively 89.44, 85.94, and 97.39% higher than those observed at 1.71 kg COD m^−3^ day^−1^. Thereafter, the organic loading rate was decreased to 0.34 kg COD m^−3^ day^−1^ from day 19 onwards, and the COD, TN, and TP removals were 89.45, 58.42, and 97.36%, respectively (Figures 4D–F). Reducing the inflow volume also affected the pollutant removal efficiencies, especially nitrogen and phosphorus. Due to a decrease in the nutrient content of the influent wastewater, nutritional imbalances might have occurred and reduced the activity of the microorganisms. The consumption of organic carbon during the aerobic phase caused a shortage of carbon source required for the denitrification process while enhancing nitrite accumulation (Kulikowska and Bernat, 2013). A C/N ratio of 6.0 was sufficient for nitrogen and phosphorus removal, while a C/N ratio <4.0 will lead to a deficiency of the carbon source for nitrogen and phosphorus removal (Renou et al., 2008). Thus, when decreasing the organic loading rate to 0.34 kg COD m^−3^ day^−1^, there was a deficit of biodegradable organic substances, thereby contributing to a decline in the nitrogen and phosphorus removal efficiencies.

Miao et al. (2015) reported that increasing the C/N ratio from 2.0 to 4.0 during landfill leachate treatment increased the nitrogen removal efficiency from 60 to 98%. Masłońa et al. (2019) ascertained that reducing the organic loading rate in synthetic wastewater from 0.68 down to 0.52 kg COD m^−3^ day^−1^ led to a decrease in the organic carbon removal efficiency from 96.7 to 93.9% and that the best organic carbon loading rate was 0.62 kg COD m^−3^ day^−1^. However, in this study, in the experiment with an organic loading rate of 2.58 kg COD m^−3^ day^−1^ (days 31–36), the TN, TP, and ammonium removal efficiencies were decreased compared to those observed at rates of 0.34 and 0.86 kg COD m^−3^ day^−1^. This behavior can be explained as due to a decline in the activity of the microorganisms caused by an organic shock load. The organic loading rate is an important parameter to be considered for bioreactor operation because an unexpected increase in the organic loading rate will cause a shock load stress and affect the structure and composition of the microbial communities, biomass-liquid separation, surface properties of the sludge, activity of the microbial community, and the pollutant removal efficiencies (Yang et al., 2018). Chelliapan et al. (2017) showed that the organic carbon rate affected the COD removal efficiency, wherein COD removal efficiencies of 99, 95, and 36.5% were obtained at organic loading rates of 0.258, 0.787, and 2.471, respectively. Singh et al. (2019) indicated that, at organic loading rates in the range of 2.55–3.15 kg COD m^−3^ day^−1^, the COD removal efficiency was in the range of 92–96% and the ammonium removal efficiency was in the range of 81–85%.

Liang et al. (2019) reported that, when the organic loading rate was increased from 1.8 to 2.5 kg COD m^−3^ day^−1^, the pollutant removal efficiency did not necessarily increase. Zhang et al. (2012) reported a COD removal efficiency of 89.8% at an organic loading rate of 1.5 kg COD m^−3^ day^−1^ of anaerobic pig manure. Zheng et al. (2017) showed that the removal of veterinary antibiotics was 85.1% at 0.17 kg COD m^−3^ day^−1^ and dropped to 75.9 and 49.3% when the COD volumetric load was increased to 0.65 and 1.07 kg COD m^−3^ day^−1^, respectively. Based on the good results achieved, it can be concluded that the optimal organic loading rate for this study is 0.86 kg COD m^−3^ day^−1^.

### Effect of C/N Ratio

The denitrification process depends on the organic carbon/nitrogen ratio (C/N ratio), and a shortage of carbon sources for the denitrification process will strongly affect the nitrogen removal efficiency. Figure 3 shows the effect of C/N ratio on the TOC removal efficiency and carbon balance. The TOC concentration in the influent wastewater and the C/N ratio did not fluctuate much, i.e., the TOC concentration was in the range of 330.12–348.56 mg/L, while the C/N ratio was in the range of 2.49–2.82. However, the TOC concentration in the effluent wastewater fluctuated from 7.33 to 61.78 mg/L. This observation clearly indicated that the low C/N ratio adversely affected the growth of microorganisms and the consumption rate of organic carbon present in the wastewater. Therefore, the optimal operation conditions are important factors in ICEAS, such as the aeration time and mixing time, to increase TOC removal efficiency.

The consumption of organic carbon in the ICEAS at an organic loading rate of 0.86 kg COD m^−3^ day^−1^ is shown in Figure 4. TOC influent = residual TOC + TOC consumed (used by the microorganisms + converted to different gas forms, e.g., CO_2_). In this study, the average residual TOC concentration was low, i.e., 26.2 mg/L. TOC removal was 92.16% compared to the TOC residual value of 7.81%, while the COD removal efficiency was 89.85% and the residual COD was 10.15%. The COD removal/TOC consumption was 6.48, and this implies that, for every 1 kg of TOC consumed, the COD removed will correspond to 6.48 kg.

### Effect of Aeration/Mixing Ratio

The effect of aeration/mixing ratio in the ICEAS was tested at aeration times of 75, 105, 165, and 195 min. The results show that the TN and TP removal efficiencies after an aeration time of 105 min (from day 43 to 48) were lower than those observed at an aeration time of 135 min (6-h cycle time). From a practical perspective, it is necessary to balance the aeration time and the mixing time in order to limit the accumulation of nitrate and convert nitrite into N_2_O and N_2_ gas. Thereafter, the aeration time was reduced to 75 min, from day 37 to 42. The results show that the TN and TP removal efficiencies sharply decreased to 60.82 and 67.90%, respectively. The average TN and TP concentrations in the effluent were 332.11 and 133.85 mg/L, respectively, which are much higher than the values observed at an aeration time of 135 min (135 min was the total aeration time in the operation cycle in the experiment to determine the optimal cycle time). The reduction of aeration time will decrease the function of the microorganisms during the conversion of ammonium into nitrate and nitrate into N_2_, resulting in the reduction of nitrogen and phosphorus removal efficiencies. Therefore, the aeration time and stirring time should be appropriately selected and maintained to achieve high performance of the ICEAS.

In the next step, the total aeration time was increased to 165 min between days 49 and 54. The removal efficiencies of TN, TOC, TP, ammonium, BOD_5_, COD, and color increased to 92.51, 94.46, 94.07, 96.65, 98.99, 94.19, and 97.06%, respectively. The treatment efficiencies at an aeration time of 165 min were higher than the values observed at an aeration time of 75 min, especially for TN and TP removal, i.e., 60.82 and 67.90%, respectively. The concentrations of COD, BOD_5_, and TN in the effluent were within the Vietnamese discharge standard values for swine wastewater effluent QCVN 62-MT:2016/BTNMT, with concentrations of 131.99, 11.62, and 65.46 mg/L, respectively. The concentrations of color, TP, TOC, and ammonium were 120.83 Pt‒Co and 24.86, 18.85, and 15.83 mg/L, respectively.

**Figure 5:**
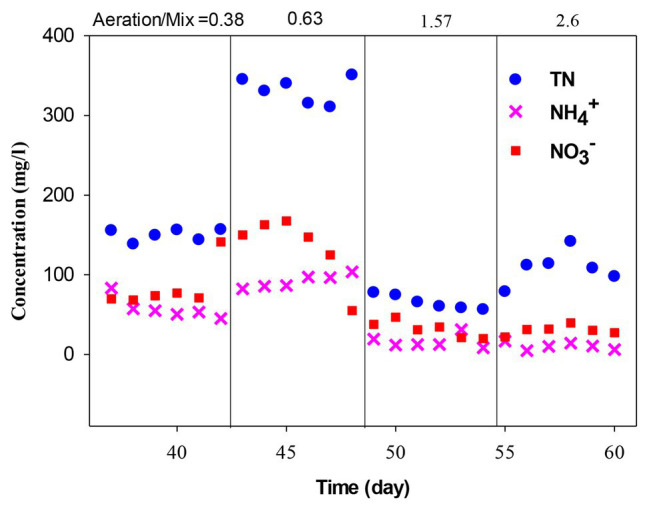
Nitrogen removal efficiency at different aeration/mixing ratios.

The bioconversion processes were carried out with sufficient oxygen concentrations required for the growth of microorganisms (Denecke et al., 2011), leading to an increase in the conversion of ammonium to nitrate. However, the aeration time of 165 min was unlikely to be optimized. Therefore, in the next experiment, the aeration time was increased to 195 min during days 55–60. The results show that the treatment efficiency after an aeration time of 195 min was nearly similar to those achieved at an aeration time of 165 min, with TN, TOC, TP, ammonium, BOD_5_, and COD removal efficiencies of 86.9, 94.9, 87.8, 97.8, 99.34, and 96.2%, respectively. From Figure 5, it is evident that an aeration/mixing ratio of 0.63 (105 min aeration/165 min mixing) resulted in ammonium, nitrate, and TN removal efficiencies of 88.16, 81.89, and 82.48%, respectively. Almost all the nitrogen present in swine wastewater was converted by the nitrification/denitrification (NH_4_^+^ → NO_2_^−^ → NO_3_^−^ → N_2_, N_2_O, N*_x_*O; Pan et al., 2014b) and partial nitrogen pathways, according to Eqs 3–6, respectively (Anjali and Sabumon, 2017).

(4) $${\mathrm{NH}}_{4}{}^{+}+1.{\mathrm{5O}}_{2}\to {\mathrm{2H}}^{+}+{\mathrm{NO}}_{2}{}^{-}+H_{2}O$$

(5) $$\begin{array}{l} {\mathrm{NH}}_{4}{}^{+}+1.31{\mathrm{NO}}_{2}{}^{-}+\\ 0.13H^{+}+0.06{\mathrm{6HCO}}_{3}{}^{-}\to 0.06{\mathrm{6CH}}_{2}O_{0.5}N_{0.15}+\\ \;\;\;\;\;\;\;\;\;\;\;\;\;2.03H_{2}O\;+0.26{\mathrm{NO}}_{3}{}^{-}+1.02N_{2} \end{array}$$

(6) $$\begin{array}{l} {\mathrm{2NO}}_{3}{}^{-}+1.25{\mathrm{CH}}_{3}\mathrm{COOH}\to N_{2}+2.{\mathrm{5CO}}_{2}+\\ 1.{\mathrm{5H}}_{2}O+{\mathrm{OH}}^{-} \end{array}$$

When this ratio was reduced to 0.38 (75 min aeration/195 min mixing), the ammonium, nitrate, and TN removal efficiencies decreased to 80.62, 70.94, and 61.82%, respectively. It is noteworthy to mention that, when reducing the aeration/mixing ratio, there is a dissolved oxygen deficit, causing a lower microbial growth rate and activity and a reduction in the pollutant removal efficiencies. The results indicate that an aeration/mixing ratio of 1.57 (165 min aeration/105 min mixing) was determined as the optimal condition, as shown in Figure 5. This also implies that nitrite accumulation is almost negligible at an aeration/mixing ratio of 1.57, and the activity and the diversity of the microorganisms were high. The ammonium, nitrate, and TN concentrations in the effluent decreased on day 51, with concentrations of 12.42, 30.98, and 65.72 mg/L, respectively. When the aeration/mixing ratio was increased to 2.60 (195 min aeration/75 min mixing), the nitrate and ammonium removals were not different compared to the values observed at a ratio of 1.57.

However, the sharp drop in TN removal efficiency suggested that increasing the aeration time will increase the microbial activity and lead to an increase in the nitrate and ammonium removal capacity and enhance the nitrite accumulation. This can also be explained by the fact that the long aeration time will promote the growth of ammonium oxidation bacteria (AOB), and this will cause a reduction in the organic carbon content. According to Xu et al. (2020), nitrogen removal from municipal wastewater in an ICEAS depends on the mixing time, and the best performance of 89% was achieved at an aeration/mixing rate of 1.5. Wang et al. (2018) indicated that an aeration/mixing rate of 1 will promote the denitrification process and improve the nitrogen removal efficiency. Song et al. (2017) carried out swine wastewater treatment at an aeration/mixing ratio of 0.63 and achieved an ammonium removal efficiency of 96.5%. Similarly, Qiu et al. (2019) performed experiments using landfill leachate wastewater in a bioreactor and reported an optimal aeration/mixing ratio of 0.5. According to Correa et al. (2018), the optimal aeration/mixing ratio was 2 when treating municipal wastewater achieved an ammonium removal efficiency of 86%. Zhang et al. (2011) treated swine wastewater using ICEAS at an aeration/mixing ratio of 2.08 and reported COD removal efficiencies >99%. The results of these previous literatures as well as the results of this study show that the optimal aeration/mixing ratio of ICEAS depends on the wastewater type and the amount of biodegradable organic compounds present in the influent wastewater. Thus, based on the results of these experiments, it can be deduced that a cycle time of 6 h, i.e., total aeration time of 165 min, mixing time of 105 min, settling time of 60 min, and a decant time of 30 min, can be considered as the optimal condition for a reliable ICEAS performance.

### Comparison With Traditional SBR

**Figure 6:**
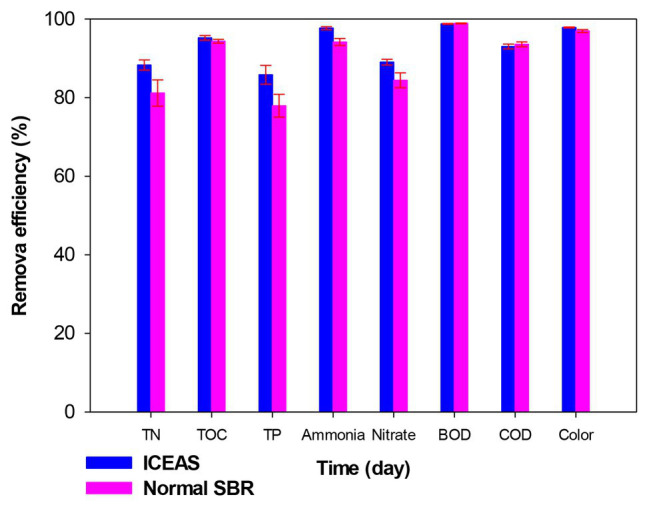
Comparison of TN, TOC, TP, ammonium, nitrate, BOD_5_, COD, and color removal efficiencies between the ICEAS and traditional sequencing batch reactor (SBR) technology.

After determining the optimal operation cycle, a comparison of the performance of ICEAS was made with a conventional SBR that was operated in parallel, under the same conditions as those of the ICEAS. According to Figure 6, the TOC (95.22%), TN (88.29%), ammonium (97.69%), TP (85.81%), and color (97.84%) removals were higher in the ICEAS than in the traditional SBR. However, the COD, BOD_5_, and TOC removal efficiencies in both reactor configurations were >90%. Khondabi et al. (2019) reported COD and BOD_5_ efficiencies of 93, and 95%, respectively, in a SBR and an ICEAS reactor. This can be explained as due to both tanks using the same activated sludge, microbial communities. Moreover, both reactors were operated at the same cycle time and hydraulic retention time, which led to having similar organic compound removal efficiencies. These results also agreed with the results reported previously by Zhang et al. (2011), wherein the nitrogen removal efficiency of ICEAS was higher than that of the conventional SBR, with values of 97 and 76.5%, respectively. Al-Rekabi et al. (2017) reported high ammonium, TP, and TN removal efficiencies of 83, 60, and 72%, respectively, in an ICEAS when compared to the conventional SBR (81, 58, and 69%, respectively) during municipal wastewater treatment. Optimizing the partial nitrification process will also help to increase the nitrogen removal efficiency of an ICEAS reactor (Ouyang et al., 1994; Zeinaddine et al., 2013). In an ICEAS, the aeration and mixing processes are time sequenced/controlled, therefore helping to maintain the desired microbial community structure and composition in the bioreactor.

### Effluent Quality and Resource Recovery Possibility

The effluent water quality of ICEAS is within the permissible values recommended by the Vietnamese discharge regulation for swine effluent (QCVN 62-MT:2016/BTNMT) that can be used for agricultural irrigation. Besides, the treated water is rich in ammonium, nitrate, and phosphate ions which are beneficial for plant growth and development. Besides, the residual activated sludge rich in phosphorus and nitrogen can be mixed with agricultural residues (e.g., coconut fibers, rice straw, and water hyacinth) to produce biofertilizers. ICEAS is a promising technology that can be applied to treat the organics and nutrients present in anaerobically digested wastewater and recover useful value-added products.

## Conclusion

The performance of an ICEAS to treat anaerobically digested wastewater was evaluated and high nitrogen and phosphorus removal efficiencies were achieved during long-term operation. The optimal cycle time was 6 h, with 165 min aeration, 105 min of mixing, 60 min settling, and 30 min of decanting time. The optimal aeration/mixing ratio was 1.57. The ICEAS was influenced by the organic loading rate (optimum value, 0.86 kg COD m^−3^ day^−1^). The performance of the ICEAS was comparatively better than that of the traditional SBR technology in terms of nutrient removal. The treated water quality was within the Vietnamese discharge standard for swine effluent (QCVN 62-MT:2016/BTNMT) for pH, COD, BOD, and TN. The ICEAS can also be applied to treat wastewaters with different physicochemical and biological characteristics, e.g., landfill leachate and industrial and municipal wastewaters. Future research should be aimed at combining/integrating the ICEAS technology with ecological treatment systems such as wetlands and waste stabilization ponds. The long-term performance of the ICEAS should also be investigated by adopting a good process control system for controlling the state variables such as pH, dissolved oxygen concentration, oxygen and reduction potential, solids retention time, and the organic loading rate. Efforts should also be made to scale up the process from a laboratory scale to a pilot and semi-industrial scale by performing suitable cost and environmental assessment studies.

## Data Availability Statement

The original contributions presented in the study are included in the article/Supplementary Material, further inquiries can be directed to the corresponding author.

## Author Contributions

ND working on experiment design. TL working on data collection and preparing the manuscript. ER support in manuscript revision. All authors contributed to the article and approved the submitted version.

### Conflict of Interest

The authors declare that the research was conducted in the absence of any commercial or financial relationships that could be construed as a potential conflict of interest.
