# Supplementary material for: Removal of Nutrients From Anaerobically Digested Swine Wastewater Using an Intermittent Cycle Extended Aeration System
Source: Front Microbiol. 2020 Oct 16;11:576438. doi: 10.3389/fmicb.2020.576438 (PMC7596319; doi:10.3389/fmicb.2020.576438)
Supplement: Supplementary file 1 [file Table_1.docx]

**Table 1.** The properties of influent swine wastewater and after ICEAS treatment.

| No | Parameter | Unit | Raw swine wastewater | After anaerobic treatment | After ICEAS treatment | Vietnamese discharge standard QCVN62-MT:2016/BTNMT |
| --- | --- | --- | --- | --- | --- | --- |
| 1 | pH | - | 6.9 | 8.4 | 6.0-7.5 | 5.0-9.0 |
| 2 | Color | Pt-Co | 4576.52 | 4104.21 | - | - |
| 3 | COD | mg/L | 3459.43 | 2267.62 | 157.78 | 300 |
| 4 | BOD_5_ | mg/L | 2100.34 | 1133.23 | 13.89 | 100 |
| 5 | TN | mg/L | 975.45 | 862.92 | 96.67 | 150 |
| 6 | NH_4_^+^ | mg/L | 623.86 | 476.35 | 10.94 | - |
| 7 | Nitrate | mg/L | 377.43 | 462.67 | 50.98 | - |
| 9 | TOC | mg/L | 389.34 | 341.18 | 26.62 | - |
| 10 | TP | mg/L | 482.62 | 415.34 | 52.46 | - |
